# A review of nitric oxide and oxidative stress in typical ovulatory women and in the pathogenesis of ovulatory dysfunction in PCOS

**DOI:** 10.1186/s12958-023-01159-6

**Published:** 2023-11-23

**Authors:** Awoniyi O. Awonuga, Olivia G Camp, Husam M Abu-Soud

**Affiliations:** 1 Departments of Obstetrics and Gynecology and Biochemistry and Molecular Biology, The C.S. Mott Center for Human Growth and Development, Wayne State University School of Medicine, 275 E. Hancock Detroit, Detroit, MI 48201 USA; 2 Department of Physiology, Wayne State University School of Medicine, Detroit, MI 48201 USA; 3 Department of Microbiology, Immunology and Biochemistry, Wayne State University School of Medicine, Detroit, MI 48201 USA

**Keywords:** Polycystic ovary syndrome, Oxidative stress, Abnormal nitric oxide pulsatility, Ano and oligo-anovulation

## Abstract

Polycystic ovary syndrome (PCOS) is a heterogeneous functional endocrine disorder associated with a low-grade, chronic inflammatory state. Patients with PCOS present an increased risk of metabolic comorbidities and often menstrual dysregulation and infertility due to anovulation and/or poor oocyte quality. Multiple mechanisms including oxidative stress and low-grade inflammation are believed to be responsible for oocyte deterioration; however, the influence of nitric oxide (NO) insufficiency in oocyte quality and ovulatory dysfunction in PCOS is still a matter for debate. Higher production of superoxide (O_2_^•−^) mediated DNA damage and impaired antioxidant defense have been implicated as contributory factors for the development of PCOS, with reported alteration in superoxide dismutase (SOD) function, an imbalanced zinc/copper ratio, and increased catalase activity. These events may result in decreased hydrogen peroxide (H_2_O_2_) accumulation with increased lipid peroxidation events. A decrease in NO, potentially due to increased activity of NO synthase (NOS) inhibitors such as asymmetric dimethylarginine (ADMA), and imbalance in the distribution of reactive oxygen species (ROS), such as decreased H_2_O_2_ and increased O_2_^•−^, may offset the physiological processes surrounding follicular development, oocyte maturation, and ovulation contributing to the reproductive dysfunction in patients with PCOS. Thus, this proposal aims to evaluate the specific roles of NO, oxidative stress, ROS, and enzymatic and nonenzymatic elements in the pathogenesis of PCOS ovarian dysfunction, including oligo- anovulation and oocyte quality, with the intent to inspire better application of therapeutic options. The authors believe more consideration into the specific roles of oxidative stress, ROS, and enzymatic and nonenzymatic elements may allow for a more thorough understanding of PCOS. Future efforts elaborating on the role of NO in the preoptic nucleus to determine its influence on GnRH firing and follicle-stimulating hormone/Luteinizing hormone (FSH/LH) production with ovulation would be of benefit in PCOS. Consequently, treatment with an ADMA inhibitor or NO donor may prove beneficial to PCOS patients experiencing reproductive dysfunction and infertility.

## Background

Polycystic ovarian syndrome (PCOS) is a heterogeneous functional endocrine disorder associated with a low-grade, chronic inflammatory state. It affects about 5–15% of women [1–3] worldwide and makes up about 70% of the ovulatory infertility cases [4, 5]. There is evidence to suggest that environmental endocrine disrupting chemicals contribute to altered fetal programming, hence, play a role in the pathogenesis of PCOS [6, 7]; however, the fundamental issue in PCOS relates to associated hyperandrogenism [8], which is supported by the discovery of naturally occurring PCOS phenotypes in non-human primates that confers a survival advantage of a hyperandrogenic and insulin resistant phenotype [8]. Such excess androgens in humans may originate from maternal, fetal, or placental sources [9, 10], and may influence circulating redox balance by regulating expression and activities of a series of cellular oxidant and antioxidative enzyme system in those with the syndrome [11, 12]. Another potential source of excess androgens arises from the high incidence of insulin resistance (IR) in PCOS, independent of obesity [13]. The increased proinflammatory state is in part due to elevation of the proinflammatory cytokine tumor necrosis factor-α (TNFα), which is a known mediator of IR [14]. Indeed, oxidative stress and IR alongside subsequent hyperinsulinemia has been speculated as a promoter of hyperandrogenism, and mitochondrial and ovulatory dysfunction in PCOS [15]. Mitochondrial mutations in PCOS [16] may lead to impaired oxidative phosphorylation, decreased adenosine triphosphate (ATP) production, and an increased production of reactive oxygen species (ROS), which may contribute to the metabolic and hormonal dysregulation in this condition by disrupting insulin signaling pathways and impairing glucose metabolism [17].

Oxidative stress is a general term used to describe the imbalance between ROS and antioxidants. It is commonly and generally applied to disease states in the body and has been shown to contribute to the biochemical parameters of PCOS. ROS are involved in redox signaling and are capable of producing molecular damage by reacting with DNA and causing mutations, carcinogenesis, apoptosis, necrosis and hereditary diseases [18]. Important and significant biomarkers of ROS-mediated DNA damage such as 8-oxoguanine (8-oxoG) and its nucleotide 8-oxo-2’-deoxyguanosine (8-OHdG) are formed when ROS react with DNA [19]. If these damaged adducts are not removed by DNA repair enzymes, their levels will increase in the tissues and reflect as low in the serum, a phenomenon that has been demonstrated in women with PCOS compared to healthy controls [20]. Kelly and collaborators [21] reported that PCOS patients exhibit chronic low-grade inflammation, manifested as elevated levels of C reactive protein. Moreover, other markers of oxidative stress such as malonodialdehyde (MDA) and advanced glycation end products (AGEs) further elucidate the advanced lipid peroxidation and metabolic dysfunction and may play an important role in IR, obesity, and reproductive dysfunction in PCOS [22–25].

The issue is further complicated because PCOS is a complex heterogeneous disorder, categorized into four phenotypes (A-D) by the revised Rotterdam Criteria [26], which entails two or three cardinal features: hyperandrogenism, ovulatory dysfunction, and PCO-like morphology of at least one ovary. Ovulatory dysfunction is a common cause of amenorrhea, abnormal uterine bleeding, and infertility [27] and in PCOS, are due to hyperandrogenemia and IR [28–30]; however, the mechanism by which they lead to oocyte and ovulatory dysfunction is still a matter for debate. Studies have shown that oxidative stress negatively affects ovarian follicles and disrupts normal follicular development and maturation [31–33]. Excessive ROS may damage oocytes and granulosa cells within the follicles, impairing their quality and compromising fertility [34, 35]. Impaired oxidative phosphorylation and mitochondrial dysfunction may contribute to IR by disrupting insulin signaling pathways and impairing glucose metabolism [36–38]. Hyperandrogenism promotes inflammation and IR, both of which can increase the production of ROS and lead to oxidative stress.

Despite the foregoing, ROS are not always harmful as they act as intracellular signaling molecules essential for immune responses and cognitive functions. Cellular sources of ROS include macrophages, neutrophils, monocytes, endothelial cells, and cardiomyocytes and from enzymes and cellular metabolism such has mitochondrial metabolism, xanthine oxidase, and cytochrome P450 [39–42]. Nitric oxide synthases (NOS), which produce nitric oxide (NO), are also potential sources for ROS under certain conditions. NO plays a functional role systemically as a freely diffusible molecule that allows for hyperpolarization and vasodilation, and as a signaling molecule that is key in several reproductive and endocrine functions. NO is generated by one of three NOS isoforms (Table 1): endothelial NOS (eNOS), inducible NOS (iNOS), and neuronal NOS (nNOS) in which L-Arginine (L-Arg) and molecular oxygen are converted into NO and L-Citrulline. Each isoform of NOS is a homodimeric hemoprotein comprised of two identical subunits each containing a bound calmodulin, requiring the cofactors zinc and tetrahydrobiopterin (H_4_B) to maintain a tight dimer and proper function [43]. Enzymatic activity can be altered due to NOS uncoupling, thereby favoring the generation of ROS such as superoxide (O_2_^•−^). O_2_^•−^ can then react with bioavailable NO to generate peroxynitrite (ONOO^−^), which contributes significantly to cytotoxicity either through induction of free radical pathways or directly through interactions with lipids, DNA, and proteins [44]. Regardless of the pathway, increased oxidative stress modulates oxidative injury to cells resulting in necrosis or apoptosis. Interestingly, recent studies have shown the role of ONOO^−^ in insulin signaling and IR, elucidating that the high inflammatory state accompanying obesity-related IR results in higher iNOS expression and thus an increase in nitration and lipid peroxidation events [45].

**Table 1:** Overview of nitric oxide synthases, reactive oxygen species, and antioxidant enzymes

Name	Origin	Function	References
Nitric oxide synthases			
Inducible NOS (iNOS)	Produced by many cell types	Essential in immune function and has major rolls in inflammatory pathology and septic shock	[165, 166]
Endothelial NOS (eNOS)	Produced in endothelial tissue	Essential for normal cardiovascular system function, blood pressure control, anti- atherosclerotic properties	[165, 166]
Neuronal NOS (nNOS)	Produced mainly in central and peripheral neurons of the central nervous system (CNS)	Essential for synaptic plasticity, central regulation blood pressure, vasodilation	[165, 166]
ROS			
Superoxide (O_2_^•−^)	Mitochondrial damage, NADPH oxidase, xanthine oxidoreductase, uncoupled NOS, NOX, cytochrome P450-dependent oxygenases, non-enzymatically, when a single electron is directly transferred to O_2_	Oxidation of proteins, lipids, and DNA; mitochondrial damage and cell death signaling; innate immune response; generation of ONOO^−^; coordination with MPO to facilitate respiratory burst to enhance chloramine and hypochlorite through H_2_O_2_ production	[39, 40, 167–169]
Peroxynitrite (ONOO^−^)	Near diffusion rate reaction of O_2_^•−^ with NO	Protein nitration; DNA damage and biomolecule modification including amino acids, proteins, enzymes, and cofactors; tyrosine nitration	[44, 167]
Hydroxyl radical (^•^OH)	Fenton reaction (H_2_O_2_ with transition metals), MPO compound II with xenobiotics substrates for cytochrome p450	Oxidative modification of amino acids, purine and pyrimidine bases of DNA, and lipids	[167, 170]
Hypochlorous acid (HOCl)	Mammalian peroxidases reaction with chloride ion and H_2_O_2_	Hemoprotein heme destruction; innate immune response with anti-microbial, anti-fungal, and anti-viral properties	[167, 170]
Hydrogen peroxide (H_2_O_2_)	Monoamine, monoacid oxidase, glucose/glucose oxidase, Superoxide dismutase, NOX4	Induction of cellular damage and arrest during cell cycle progression; facilitation of cell death; promoter for cell cycle progression	[39, 167, 170]
Antioxidant enzymes			
Superoxide Dismutase (SOD)	Cytosolic copper/zinc-SOD, mitochondrial manganese-SOD, and extracellular SOD	Catalyzes the dismutation of the superoxide anion to O_2_ and to the less reactive species H_2_O_2_	[39, 169, 171]
Catalase (CAT)	Mammalian catalase is present in peroxisomes	Reacts with H_2_O_2_ to form water and molecular oxygen and reacts with H donors (methanol, ethanol, formic acid, or phenols) with peroxidase activity; protects cells from self-generated H_2_O_2_	[39, 171]
Glutathione peroxidase (GP)	Cytosolic and mitochondrial glutathione peroxidase (or GPX1) is found in most tissues and in erythrocytes, kidney, and liver. The phospholipid hydroperoxide glutathione peroxidase (GPX4) are found in most tissues and is highly expressed in renal epithelial cells and testes. Cytosolic GPX2 and extracellular GPX3 are found in the gastrointestinal tract and kidney, respectively.	Catalyzes the reduction of fatty acid hydroperoxides and H_2_O_2_ using glutathione (GSH)	[171]

NO has been proposed to prevent atresia and apoptosis in developing follicles [46–50] and lowered level of NO is reported in women with PCOS [51]. However, there is evidence to suggest that estrogen stimulated gonadotropin releasing hormone (GnRH) secretion could be mediated via increased NO production in the median eminence [52]. In addition, research applying chemistry and biochemistry suggest that aside from biologically active proinflammatory mediators, metallic compounds also have a role in the pathophysiology of PCOS [93]. Notable among these metals is zinc, which is an essential cofactor and a signaling ion for NOS dimeric activity [43]. Further, given that anovulation is ranked as the most common cause of infertility with low documented IVF/ICSI success rates [3], it is incumbent on scientist to study this phenomenon in PCOS [1] (see Table 2 for current studies on NO and PCOS and oxidative stress and PCOS). The objective of this review is to connect the current research findings in PCOS and the contribution of NO/NOS in attempts to explain its associated ovulatory dysfunction. We hypothesize that altered enzymatic activity and distribution of ROS promotes a deficiency in H_2_O_2_ and NO that may facilitate the disruption of the ovulatory process in PCOS, including steroidogenesis, oocyte maturation, and cumulus cell expansion.

**Table 2:** PCOS studies with nitric oxide and oxidative stress

Topic with PCOS	Study Title	Reference
Nitric oxide	Nitric oxide (NO) levels in patients with polycystic ovary syndrome (PCOS): a meta-analysis	Meng C [134].
	Clomiphene citrate increases nitric oxide, interleukin-10 and reduces matrix metalloproteinase-9 in women with polycystic ovary syndrome	Sylus AM et al., [172]
	Impaired Arginine Metabolism Coupled to a Defective Redox Conduit Contributes to Low Plasma Nitric Oxide in Polycystic Ovary Syndrome	Krishna MB et al., [51]
	Nitric oxide donors improve the ovulation and pregnancy rates in anovulatory women with polycystic ovary syndrome treated with clomiphene citrate: A RCT	Mahran A et al., [112]
	Cardiac Nitric Oxide Synthases and Na+/K+-ATPase in the Rat Model of Polycystic Ovary Syndrome Induced by Dihydrotestosterone	Tepavčević S et al., [173]
	Detailed characterisation of circulatory nitric oxide and free radical indices–is there evidence for abnormal cardiovascular homeostasis in young women with polycystic ovary syndrome?	Willis GR et al., [174]
	Assessment of paraoxonase 1, xanthine oxidase and glutathione peroxidase activities, nitric oxide and thiol levels in women with polycystic ovary syndrome	Baskol G et al., [158]
	Polymorphisms of the endothelial nitric oxide synthase gene in premenopausal women with polycystic ovary syndrome	Walch K et al., [175]
	Nitric oxide and fibrinogen in polycystic ovary syndrome: associations with insulin resistance and obesity	Nácul AP et al., [176]
Oxidative stress	The interplay of oxidative stress and immune dysfunction in Hashimoto’s thyroiditis and polycystic ovary syndrome: a comprehensive review	Batóg G et al., [177]
	Heavy Metals and Essential Elements in Association with Oxidative Stress in Women with Polycystic Ovary Syndrome-A Systematic Review	Srnovršnik T et al., [178]
	Efficacy of omega-3 polyunsaturated fatty acids on hormones, oxidative stress, and inflammatory parameters among polycystic ovary syndrome: a systematic review and meta-analysis	Yuan J et al., [179]
	Influence of n-3 fatty acid supplementation on inflammatory and oxidative stress markers in patients with polycystic ovary syndrome: a systematic review and meta-analysis	Tosatti JA et al., [180]
	Oxidative Stress and Polycystic Ovary Syndrome: A Brief Review.	Mohammadi M [12]
	The Effects of Probiotic Supplementation on Clinical Symptom, Weight Loss, Glycemic Control, Lipid and Hormonal Profiles, Biomarkers of Inflammation, and Oxidative Stress in Women with Polycystic Ovary Syndrome: a Systematic Review and Meta-analysis of Randomized Controlled Trials	Tabrizi R et al., [181]
	The Effects of Vitamin D Supplementation on Biomarkers of Inflammation and Oxidative Stress Among Women with Polycystic Ovary Syndrome: A Systematic Review and Meta-Analysis of Randomized Controlled Trials	Akbari M et al., [182]
	Circulating markers of oxidative stress and polycystic ovary syndrome (PCOS): a systematic review and meta-analysis	Murri M et al., [183]
	Polycystic Ovary Syndrome and Oxidative Stress-From Bench to Bedside.	Zeber-Lubecka; et al., [17]
	The Silent Threat to Women’s Fertility: Uncovering the Devastating Effects of Oxidative Stress	Kaltsas A et al., [184]
	A brief insight into the etiology, genetics, and immunology of polycystic ovarian syndrome (PCOS)	Siddiqui S et al., [28]
	Reactive oxygen species in reproduction: harmful, essential or both?	Jamil M et al., [185]
	Mitochondrial function in women with polycystic ovary syndrome	Cozzolino M & Seli E [186]
	Applications of Melatonin in Female Reproduction in the Context of Oxidative Stress	Jiang Y et al., [187]
	Oxidative stress in oocyte aging and female reproduction	Wang L et al., [188]
	Controlling chronic low-grade inflammation to improve follicle development and survival	Yang Z et al., [189]
	A novel and compact review on the role of oxidative stress in female reproduction	Lu J et al., [190]
	Source and amount of carbohydrate in the diet and inflammation in women with polycystic ovary syndrome	Barrea L et al., [191]
	Impact of stress on female reproductive health disorders: Possible beneficial effects of shatavari (Asparagus racemosus)	Pandey AK et al., [192]
	Oxidative Stress in Granulosa-Lutein Cells From In Vitro Fertilization Patients	Ávila J et al., [193]
	Oxidative stress and cardiovascular complications in polycystic ovarian syndrome	Hyderali BN & Mala K [115]
	The effects of oxidative stress on female reproduction: a review.	Agarwal A et al., [194]

### Methodology

An extensive literature review was conducted through the online databases PubMed, Science Direct, and Springer Link up to March 2023, using the keywords “polycystic ovary syndrome/PCOS”, “nitric oxide”, “M1/M2 macrophages”, “arginase”, “asymmetric dimethylarginine/ADMA”, “oocyte development”, “oocyte maturation”, “reactive oxygen species/ROS”, “anovulation”, and “antioxidants”. References included in this work are peer reviewed articles written in the English language, and references in the retrieved articles were individually hand searched for additional related references. Original articles were selected presenting an overview of PCOS, oligo-anovulation, anovulation, inflammation, and ROS/oxidative stress with infertility.

## Ovulation in normal menstruating women

**Fig. 1:**
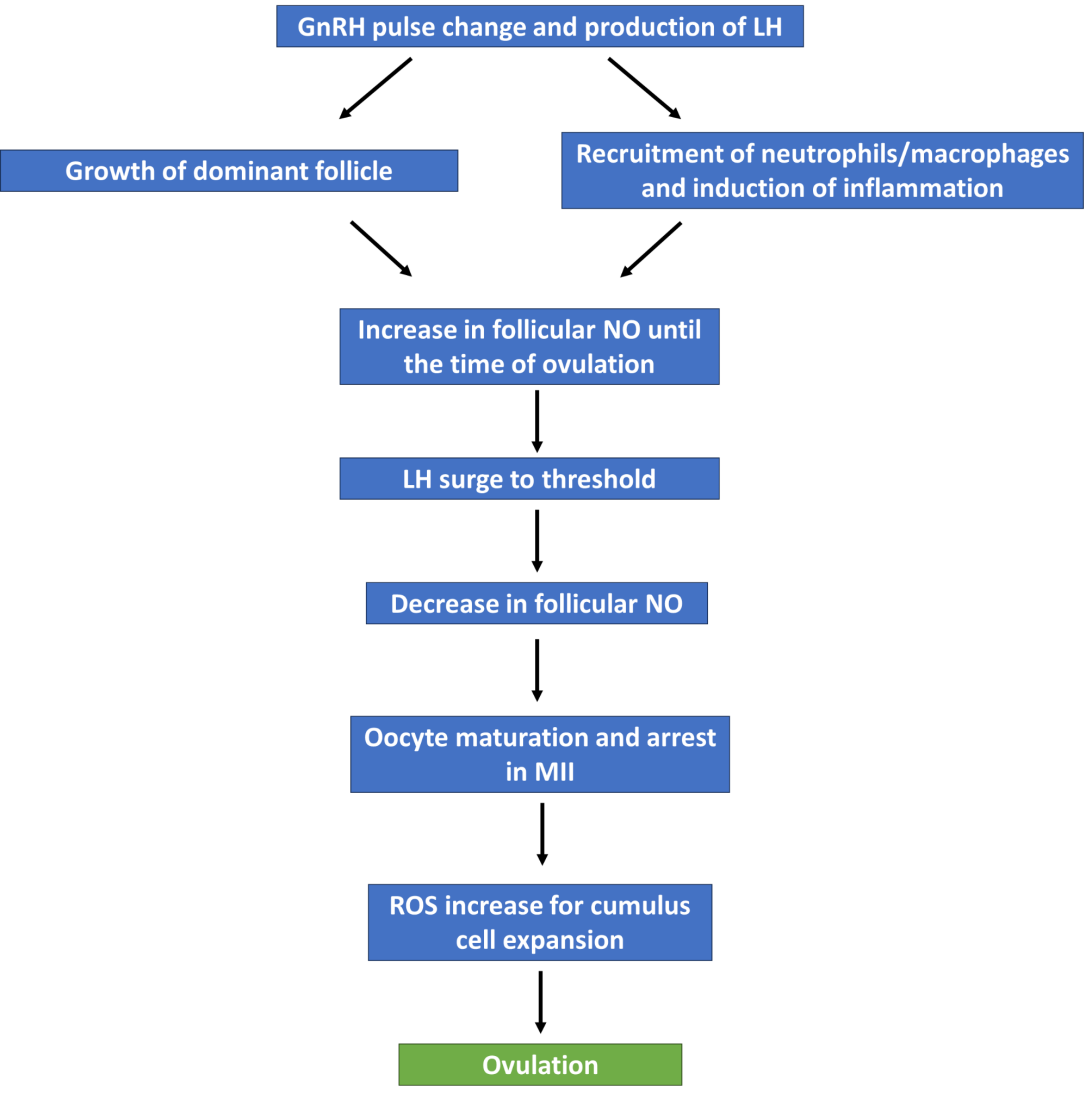
Physiological requirements for the induction of ovulation

Females are born with a finite number of oocytes, about 200,000- 600,000. These oocytes are arrested at the diplotene stage of prophase I of the first meiotic division as a germinal vesicle (GV) in primordial follicles, which contain primary oocytes until puberty and before the start of each ovulatory cycle. Two nuclei in the brain are important as it relates to ovarian function: the thalamus and the hypothalamus. Ovulation requires a series of events, beginning with coordinated firing of GnRH neurons (Fig. 1) by the preoptic area within the hypothalamus as it relates to frequency and amplitude [53] for sex hormone regulation. NO production occurs in the preoptic nucleus in close proximity to cell bodies of GnRH-immunoreactive neurons. The rostral preoptic hypothalamic areas are rich in nNOS gene expression, as seen by in situ hybridization [54, 55]. This is supported by a recent study by McCosh et al., [56] that uses a sheep model to produce data to support the hypothesis that the population of somatostatin neurons in the ventral lateral region of the ventral medial nucleus of the thalamus are a source of NO as they contained nNOS. These neurons synapse onto GnRH neurons and neurons co-expressing kisspeptin, neurokinin B, dynorphin. The GnRH neurons release GnRH into the tuberoinfundibular tract and is transported through the hypophyseal portal system to the gonadotrophs in the anterior pituitary gland where they synapse on the GnRH receptors to cause the coordinated release of pituitary gonadotropins (follicle stimulating hormone (FSH) and luteinizing hormone (LH)). The firing of these neurons is increased during the LH surge compared with other phases of the menstrual cycle hence represent an important site of estradiol (E2) positive feedback. To confirm these assertions, the authors [56] also showed that intracerebroventricular infusion of the NOS inhibitor, N(G)-nitro-L-arginine methyl ester, completely blocked the estrogen-induced LH surge.

At each ovulatory menstrual cycle, a select group of primordial follicles are recruited. The rate of firing of the GnRH pulse generator – a highly calcium-dependent and cyclic adenosine monophosphate (cAMP) stimulated phenomenon [57, 58] - determines which gonadotropin is synthesized by the preoptic nucleus whereby more rapid pulses of GnRH neurons preferentially increase synthesis and secretion of LH. FSH is preferentially stimulated by slower-frequency GnRH pulses [59, 60] that start in the luteal phase of the previous menstrual cycle leading to production of FSH [53] in the early follicular phase. This rise in FSH allows the recruited primordial follicles to begin growing, with one eventually becoming the dominant follicle. These processes are tightly regulated by intrafollicular events including paracrine factors in the theca, mural, and granulosa cells with a heavy reliance on oocyte competence suggesting a potential “checkpoint” in the process [61–63]. In ovulatory women, the follicle that has grown to acquire the highest amount of FSH receptors continues to respond to the falling FSH as the cycle progresses while the remaining unrecruited follicles undergo apoptosis. High concentration of NO is associated with apoptotic cell death, which is supported by an increase in cell apoptosis following induction of iNOS and use of exogenous NO donors [64] suggesting that NO may be involved in apoptosis of non-dominant follicles. In the mid follicular phase, the GnRH pulse generator firing changes to increased frequency and amplitude of LH production. By this time, the surface of theca and granulosa cells of the dominant follicle express more LH receptors rather than FSH receptors that are responsible for the notable shift in the ovarian follicle steroidogenic pathway mediated by membrane-bound G protein-coupled receptors localized on the surface of follicle cells [65]. This increase in GnRH firing leads to a rise in plasma LH that drives the transition from follicular growth to maturation.

The surge in LH has been shown to initiate several changes in the dominant follicle microenvironment that culminate in follicular rupture and ovulation. The LH surge also initiates the increase in the production of cAMP, steroidogenesis, and the release of inflammatory mediators that promote angiogenesis and hyperemia to degrade the follicle’s connective tissue in preparation for ovulation [60, 66, 67]. NO is also believed to be involved in follicular development as its level increases during follicle growth and decreases immediately after ovulation [47].

Macrophages are key in the inflammatory response and are the most abundant immune cells in the ovaries with important functions in ovarian homeostasis [68]. Similarly, intraovarian macrophages vary in their location and distribution during different stages of the cycle, and are present in peri-ovulatory human follicular fluid, suggesting that macrophages and macrophage derived products play an important regulatory role in intra-ovarian events including folliculogenesis, tissue restructuring at ovulation, and corpus luteum formation and regression [69]. Further, given that ROS originate from inflammatory cells such as macrophages and neutrophils, which are known to be recruited to the ovary following the LH surge, the induction of inflammation by the LH surge elucidates the participation of ROS. [46, 47]. Shkolnik and collaborators [66] provided strong evidence that ovulatory response is associated with inflammation, and involvement of ROS. The authors showed that administration of scavengers of oxidative species into the ovarian bursa of mice hormonally induced to ovulate, significantly reduced the rate of ovulation while LH-stimulated up-regulation of genes, crucial for ovulation, was substantially attenuated upon ROS ablation. These authors also showed that antioxidants prevented LH-induced cumulus expansion, necessary for ovulation, and caused impaired progesterone production in isolated follicles incubated with LH [66].

Finally, following ovulation, the follicular remnant forms the corpus luteum, that contains luteinized granulosa cells responsible for progesterone synthesis in preparation for a possible pregnancy [70]. If pregnancy does not occur, the drop in LH after ovulation alters the frequency of GnRH release, restarting the secretion of FSH and ensuing the next menstrual cycle.

## Ovulatory dysfunction in PCOS

In patients with PCOS, GnRH pulse frequency and amplitude are persistently increased [71] favoring the production of high plasma LH relative to FSH, which remains in the low levels seen in the early follicular phase, a typical phenomenon seen in combination with anovulation, and the arrest of antral follicles. The mechanisms responsible for the neuroendocrine abnormalities in PCOS is still not well elucidated, however, studies have revealed decreased sensitivity of the GnRH pulse generator to inhibition by ovarian steroids (estradiol and progesterone) [61–63] may be at play. In addition, MiRNAs [72], mutations and SNPs of nuclear-encoded genes (such as FSHR, LHCGR, and others) have been linked to PCOS development and pathogenesis [73, 74].

Three out of the four phenotypes of PCOS according to the Rotterdam Criteria include ovulatory dysfunction characterized by chronic anovulation. This phenomenon in PCOS occurs with (type A and B phenotypes) or without (Type D phenotype) elevated androgens. Notably, there are no clear androgen levels that definitively classify biochemical hyperandrogenism in PCOS, and serum androgens can be affected by metabolic state [75]. In addition, it is accepted that the hyperandrogenemia in PCOS can be clinical only [76], all of which add to the complexity of the disorder. In PCOS patients, typically, there is increased estradiol secretion from the ovary, a continuous high frequency pulse generation of GnRH, decreased FSH, and a persistently high LH level that does not reach the threshold LH “surge” that typically would induce ovulation [70, 77]. Further, it is common to see estradiol (E2) levels within a normal range expected during the early to mid-follicular phase of a menstrual cycle [78–80].

The chronic anovulation plus hyperandrogenemia in PCOS have been linked to chronic low-grade inflammation. The increase in low grade chronic inflammation in PCOS is associated with increase macrophage infiltration. Macrophages express the enzymes myeloperoxidase (MPO) and arginase, and may both contribute to a decrease in NO bioavailability [69] in PCOS similar to what entails in ovariectomized pigs [81] and after oophorectomy [82]. Although there are conflicting reports regarding increased inflammatory markers such as C-reactive protein and alteration in the expression of inflammatory cytokines [e.g., upregulation of interleukin (IL) 6, IL8, IL1β, and TNFα] in granulosa cells in PCOS [83–88], there is evidence they may result in a premature influx of leukocytes, specifically through macrophage activation. To support these assertions, metformin and troglitazone used in PCOS (an agonist of the peroxisome-proliferator-activated receptor gamma), with insulin sensitizing abilities in adipocytes have been shown to exert potent anti-inflammatory effects in macrophages [81, 82] that not only improve insulin sensitivity but also reduces inflammation, measured as C-reactive protein levels [89].

## NO and ROS in oocyte development and ovulation in normal menstruating women

NO functions to inhibit aromatase, an enzyme that works to convert androgens to estrogens [90], in the ovary [91]. During the menstrual cycle the rise in estrogen, namely 17 beta-estradiol (E2), signals the change in pulse frequency of GnRH resulting in decreased FSH and increase LH secretion [92, 93]. The surge in LH then signals meiotic maturation and the beginning of ovulatory events. NO is reported to be crucial for induction of the LH surge in mammals, with studies focusing on rats and, more recently, sheep [56, 94–96]. In ovariectomized rats, Bonavera and his colleagues [95] determined that the magnitude and duration of the LH surge is lower than in wild type cycling rats potentially because there is a low-grade stimulatory feedback of E2 on LH secretion. Moreover, they determined treatment with L-Arg, essential for NOS function, enhances the LH surge in E2-primed ovariectomized rats [95] signifying the role of NO in the physiological ovulatory process (Fig. 1). First, the LH surge results in a decrease in follicular iNOS and NO, and subsequently cyclic guanosine monophosphate (cGMP) through phosphorylation of gap junction proteins, namely connexin-43, between the granulosa cells [39–41]. Cyclic guanosine monophosphate mediates physiological functions of NO, such that when cGMP is high it antagonizes the activity of phosphodiesterase 3 A (PDE3A), promoting oocyte arrest [42]. The decrease in cGMP following the surge in LH subsequently increases PDE3A activity, that hydrolyzes cAMP allowing for germinal vesicle breakdown (GVBD) and formation of the first meiotic spindle in metaphase I [42]. The oocyte can then resume meiosis and progress until its arrest in metaphase II following the release of the first polar body at the time of ovulation, ready to be fertilized [43]. Second, the LH surge increases the activity of the proteolytic enzymes that weaken the ovarian wall thus allowing for extrusion of the oocyte [36]. These processes are in keeping with studies that investigated the role of oocyte quality in ovulation, determining that poor oocyte quality or oocyte-cumulus miscommunication results in anovulatory events. In mice lacking connexin-37, an essential gap junction protein, for example, large preovulatory follicles develop but do not produce resultant ovulation [44]. Similarly, damage or deletion to gene products necessary for oocyte chromatin modifications can cause a reduced ovulation rate [45]. Others also concurred [97–99] and showed that inhibition of iNOS in rats results in a reduction of ovulation rates by 50%, an outcome that is reversed by treatment with an NO donor. Another study showed NO enhances vasodilatation, which is responsible for follicle selection and maturation in both spontaneous and stimulated *in-vitro* fertilization cycles [109, 110]. However, it is important to note that research regarding cyclical fluctuations of NO in regard to fertility is lacking. A study by Mandhane et al. [100]., investigated exhaled NO parameters during the menstrual cycle as a way to determine the cyclical effects previously observed in asthma and atopy of normally cycling women. They concluded exhaled NO decreased during phases of the menstrual cycle that correlated with increased estrogen, whereas when progesterone levels were high there was an increase in exhaled NO. Intuitively, the functions of NO can be speculated to increase vascular flow to the ovary and developing follicle while also allowing for proper buildup of the uterine lining during the follicular phase of the menstrual cycle. One study [101], found that in fertile women, NO metabolites were higher during the follicular phase compared to the secretory phase with levels reaching a maximum at midcycle. Similarly, a study by Ota et al. [102]., found the expression of eNOS in the endometrium gradually increased beginning at the early proliferative phase through the mid-secretory phase, in which levels were greatest.

## NO, ROS, and ADMA in oocyte development and anovulation in PCOS

### NO in oocyte development and anovulation in PCOS

Patients with PCOS recruit more than the average number of antral follicles as ascertained by baseline early follicular phase transvaginal ultrasound [103]. At the level of the oocyte, NO plays a role in meiotic maturation [48, 49], and mediates an anti-apoptotic effect preventing premature atresia of developing follicle. Importantly, the deficiency of NO and H_2_O_2_ in the follicular microenvironment of PCO follicles may have substantial impacts on development of oocytes therein and ovulation. In support of this assertion is one study that showed intra-follicular milieu in NO treated patients improved follicular growth, oocyte quality and maturation [104]. Therefore, reduced NO seen in PCOS may be associated with arrest of follicular development. This is in keeping with another experiment, this time in rats that found specific iNOS inhibitor aminoguanidine inhibited ovulation by 50%, an effect that was completely reversed by NO donor sodium nitroprusside [99]. Similarly, the relative increase estradiol production in PCOS is akin to experiments that showed NO inhibition caused constant estrous in rats [105]. Without NO and H_2_O_2_, it is likely there will be altered estrogen signaling, arrested follicles, immature oocytes, inadequate cumulus-cell expansion, and decreased ovulatory events.

As mentioned previously, NO is essential for the production of cGMP and it does this through its role as a ligand of soluble guanylyl cyclase. Soluble guanylyl cyclase catalyzes the conversion of GTP to cGMP, thus NO is required to maintain cGMP levels and oocyte arrest [106–109]. Although NO is low in PCOS, Fan et al., [110] found elevated concentrations of cGMP in PCOS patients suggesting this ligand may play a role in follicular arrest in PCOS and hence its associated chronic anovulation; therefore, data regarding GMP in this syndrome remain controversial and it is possible that elevated concentration of cGMP in PCOS is through another but unknown mechanism. Afterall, increase cGMP without associated increase NO is seen with use of potassium channel openers, for example Nicorandil, known to open ATP-sensitive potassium (K-ATP) channels, can elevate cGMP levels in some tissues, without direct NO generation [111]. It seems, therefore, that any mechanism that would decrease the expression of cGMP and cAMP would activate meiotic resumption and hence increase the rates of metaphase II (MII) oocytes and eventually ovulation. One small RCT [112] evaluated the effect of isosorbide mononitrate (ISMN), a NO donor on the ovulation and pregnancy rates in 90 anovulatory women with PCOS randomly allocated into three 5-day clomiphene citrate (CC) treatment groups namely; 100 mg CC only, and with additional intravaginal 10 mg or 20 mg of ISMN respectively. The authors reported significant increase in the ovulation and pregnancy rates in the patients treated with CC + ISMN as compared with patients treated with CC alone (p < 0.001). These are yet another evidence that the chronic anovulation seen in anovulatory PCOS phenotypes may be due to decrease NO production.

### The role of ADMA, a NOS inhibitor, in the pathogenesis of decreased NO production in PCOS

**Fig. 2:**
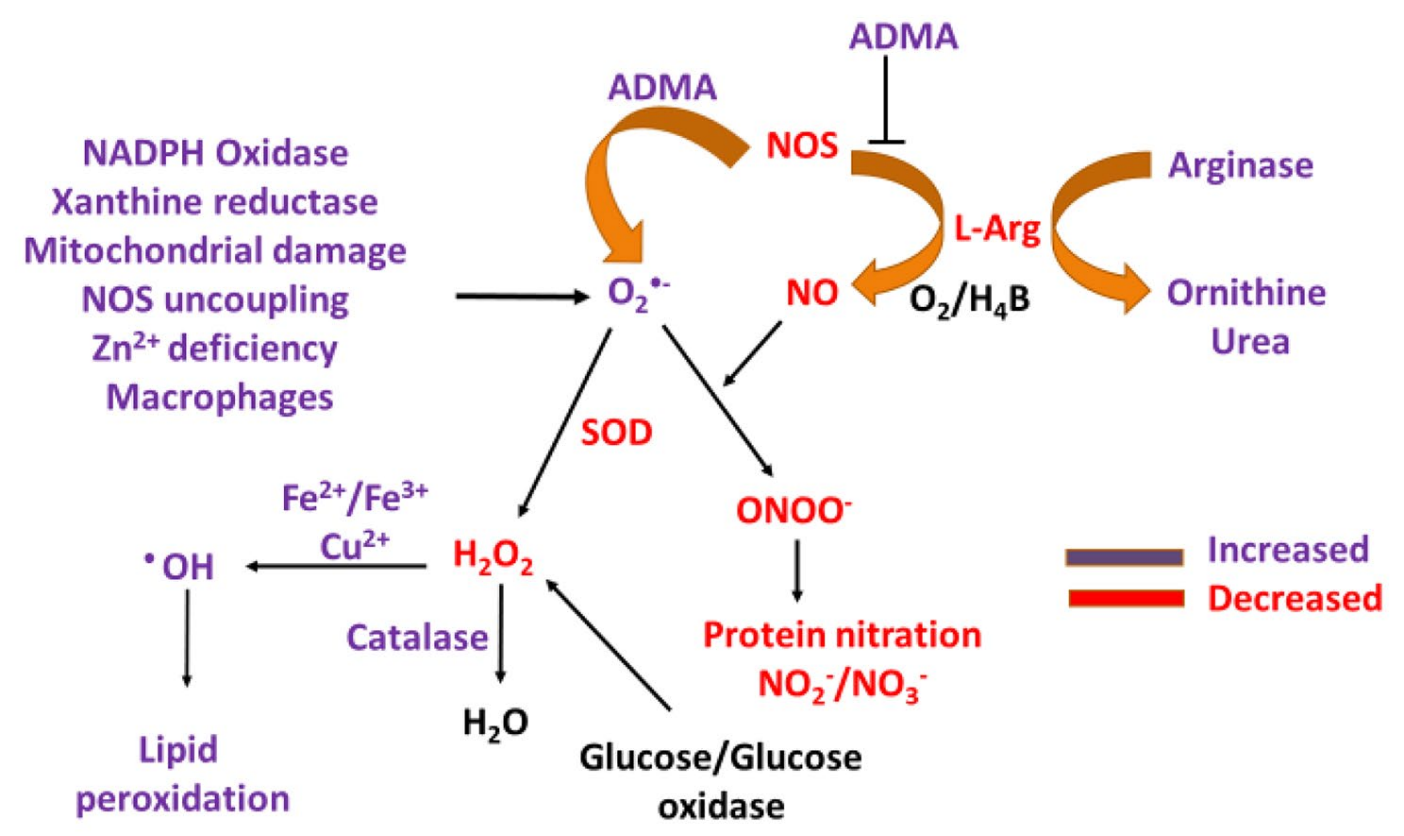
Simplified model outlining NOS dysfunction and modulation of ROS in PCOS- High concentrations of ADMA functions to competitively inhibit NOS at the L-Arg binding site, resulting in O_2_^•−^ and free L-Arg accumulation. The accumulated L-Arg may be consumed under these conditions by arginase to give ornithine and urea. Other sources for O_2_^•−^ are NADPH oxidase, xanthine reductase, mitochondrial damage, zinc deficiency, and high macrophage activity, which may be increased in PCOS. O_2_^•−^ without sufficient NO will not produce ONOO^−^ resulting in low observed protein nitration. Accumulation of O_2_^•−^ either slowly decays to H_2_O_2_ or in the presence of sufficient zinc is dismutased by SOD into H_2_O_2_. The low reported zinc concentrations and high copper in PCOS suggests low SOD activity. H_2_O_2_ then is converted to H_2_O by catalase and/or reacts with free metals such as iron and copper through the Fenton reaction to generate the highly toxic ^•^OH, resulting in lipid peroxidation events

Asymmetric dimethylarginine (ADMA), a known endogenous competitive NOS inhibitor plays a role in the pathogenesis of decreased NO production in PCOS [113] (Fig. 2). ADMA functions to competitively bind to the L-Arg site of NOS, disrupting the function of the enzyme resulting in ADMA induced O_2_^•−^ production [43]. Akedmir and colleagues reported that serum ADMA levels have small fluctuations throughout the menstrual cycle, with levels increasing in the follicular phase and decreasing in the luteal phase [114], and ADMA has been noted to be elevated in the plasma of individuals with PCOS [115] as well as in some of its other sequela such as hypercholesterolemia, hypertension and atherosclerosis, all of which are associated with reduced NO synthesis [116–119]. Using Dehydroepiandrosterone (DHEA)-induced PCOS Sprague Dawley rats and the ovarian granulosa cell line KGN, Li and colleagues [115] investigated the effect of the ADMA-dimethylarginine dimethylaminohydrolase 1 (DDAH1) pathway on redox status and ovarian apoptosis. These rats were noted to have higher ADMA levels in serum and lower DDAH1 expression in their ovaries. ADMA treatment of the KGN cells induced ROS accumulation and led to apoptosis. Overexpression of DDAH1 enhanced cell viability, and inhibited oxidative stress, while the effect was reverse in DDAH1 knockdown cells. These authors [115] also quantified the ADMA levels and redox status in serum specimens of 19 women with PCOS and 17 healthy women (controls) and showed that women with PCOS had increased serum ADMA levels and decreased glutathione peroxidase (GSH-PX) compared with the controls. These experiments demonstrate the involvement of elevated ADMA levels and redox imbalance in PCOS, which would suggest that alterations in the activity of DDAH could interfere with NO concentrations by increasing or decreasing ADMA [120].

In the follicular fluid obtained from women participating in an IVF program, Bódis et al., [120] noted that elevated levels of L-Arg and methylarginines have adverse effect on the number of oocytes and embryos generated, thus negatively impacting reproductive function. These authors reported the mean ADMA levels in the FF was 0.470 µM in those with less than 6 developed embryos and 0.368 µM in those with greater than 6 developed embryos. Given that ADMA is known to be increased in PCOS, it is plausible that enhanced ADMA may inhibit NOS functionality in this disorder, leading to a decrease in NO production as well as associated harmful sequela such as hypertension, obesity, and IR [121–124].

### The competition between NOS and arginase in PCOS

Arginase is the last step enzyme in the hepatic urea cycle, but recent research has identified its role in normal and several other pathophysiological processes [125]. In PCOS, there is enhancement of arginase levels and alteration in arginine metabolism. Macrophages traditionally generate arginase, which is a metalloenzyme found in mammals in two forms: arginase-I (Arg-I), primarily in the cytoplasm, and arginase-II (Arg-II), primarily in the mitochondrion [126, 127]. Androgen stimulation was found to upregulate interleukin-8 (IL-8) and that it directly increased the expression of Arg-I and Arg-II [128]. Both isoforms hydrolyze L-Arg to generate urea and L-orinthine. Arg-I is associated with expression in M2 macrophages in which it competes with iNOS, generated by M1 macrophages, thereby reducing iNOS activity and NO concentration, while Arg-II influences the macrophage inflammatory response by promoting mitochondrial ROS such as O_2_^•−^ [119]. There have been conflicting reports surrounding the ability of arginase to compete with NOS for L-Arg due to kinetic studies elucidating low-affinity for binding in biological systems [129–131]; however, a recent kinetic simulation model by Momma and Ottaviani [129] that aimed to investigate the competition between the enzymes for L-Arg concluded that even under extreme conditions where the Vmax ratio is 100,000:1 (arginase:NOS), arginase does not outcompete NOS. Under conditions in which ADMA activity is increased as with PCOS, the competitive inhibition through the L-Arg binding site may decrease the competition between NOS and arginase allowing arginase to consume the free L-Arg. This pathway may explain the enhancement of arginase levels and alteration in arginine metabolism seen in PCOS leading to the commonly observed increased ornithine levels [132, 133]. Kyselova and collaborators [133] found that the ratio of ornithine to arginine was significantly increased in plasma from PCOS patients and was associated with a significant increase in plasma arginase levels and activity compared to control. Further, this theory is in agreements with the work done by Krishna et al., [51] who in a retrospective cohort study analyzed NO_2_^−^/NO_3_^−^ and H_2_O_2_ concentrations, transcript levels of endothelial NOS (eNOS)/iNOS, arginine modulators, and H_2_O_2_ regulators in PCOS women (N = 29) and non PCOS controls (N = 20). The authors conclude that PCOS women have lowered NO due to reduced levels of iNOS/eNOS expression, low H_2_O_2_, high ADMA synthesis and reduced arginine bioavailability. Moreover, the reduction in H_2_O_2_ may be due to increase in catalase levels, a consequence of the body’s effort to alleviate the oxidative burden in the system (Fig. 2). Therefore, arginine bioavailability may play an important role in ovulatory dysfunction and oocyte quality, as the functionality of NOSs are disturbed. Lastly, studies including a metanalysis show decreased serum or plasma serum nitrate/nitrite in this disorder, meaning low protein nitration [134].

## Metallic compounds, proinflammatory mediators, SOD dysfunction and distribution of ROS in PCOS

It is known that women with PCOS are deficient in several minerals such as zinc, magnesium, calcium, and potassium [109, 110, 135]. In particular, zinc, an essential cofactor and a signaling ion for NOS dimeric activity [43] is necessary for the oocyte to form a fertilization-competent egg through its anti-inflammatory, anti-apoptotic, and antioxidant properties [136–138]. Serum zinc concentrations has been reported low (Fig. 2) in patients with PCOS [139–141], which may drive NOS dysfunction and a deprivation of NO bioavailability. Further, Lai [116] and colleagues demonstrated in porcine oocytes that zinc was a critical trace mineral for maintaining oocyte quality by regulating mitochondrial function and autophagy. Insufficient zinc and/or H_4_B can cause alterations to NOS dimeric functioning [43] and impaired antioxidant defense [115, 117, 118] resulting in detrimental effects to NO levels, spindle/chromatin integrity, and oocyte quality.

Notably, when O_2_^•−^ activity is increased it may reduce NO bioavailability through a reaction resulting in the production of ONOO^−^. ONOO^−^ may then disturb the cysteine residues in the zinc cluster of NOS resulting in the release of zinc and further modification of NOS functionality, NO deficiency, and oxidative stress generation [43, 142]. O_2_^•−^ may also undergo a nonenzymatic or SOD-catalyzed reaction to generate H_2_O_2_, and both can contribute to the production of proinflammatory cytokines, by monocytes, and macrophages [143–145] and can participate in the consumption of NO. It is of note that SOD has been found to be lower in PCOS patients compared to control [146]. Two forms of SOD bind to zinc, the first is copper/zinc SOD (CuZnSOD or SOD1), and the second is extracellular SOD (ECSOD or SOD3) which also binds Cu and Zn. Significantly lower activity of SOD1 and Cu/Zn concentration were found in a group of women with PCOS compared to control [147]. Insulin resistance in PCOS women causes further decrease in SOD1 activity, while Cu concentration and the value of Cu/Zn was increased when compared to women with normal insulin levels [147]. SOD1 is present in the cytosol, nucleus, peroxisomes, and in small amounts in the mitochondrial membrane of cells and acts to lower the steady-state concentration of O_2_^•−^ by dismutating it to H_2_O_2_ [148]. Once the SOD enzymes dismutase O_2_^•−^ to H_2_O_2_, enzymes such as glutathione peroxidase, peroxiredoxins, and catalase, enzymatically convert H_2_O_2_ to water [149–151], which may explain the low reported accumulation of H_2_O_2_ in PCOS [152]. Zinc deficiency or mutations in SOD1 resulting in a zinc deficient enzyme may disrupt SOD1’s function creating a shift to prooxidant activity [148, 153–155]. Conversely, if H_2_O_2_ production increases and is not removed promptly by antioxidants it can generate the more cytotoxic hydroxyl radical (^•^OH) that contributes to lipid peroxidation [104, 156] (Fig. 2) known to be associated with PCOS through reaction with trace amounts of transition metals such as iron, cobalt and copper either by the Fenton reaction or Haber-Weiss reactions [157]. In a prospective case control study, Baskol and colleagues [158] reported that serum xanthine oxidase (XO) (a generator of ROS) activities were higher in women with PCOS than in control women while and antioxidant status is decreased as ascertained by decreased lipid antioxidant paraoxonase 1 (PON1) activity [158]. This would suggest that PCOS women are under oxidative stress with resultant XO-mediated lipid peroxidation. This can further be proven through the reported increase in MDA, a marker of lipid peroxidation, in patients with PCOS [23, 159]. The associated increase in lipid peroxidation may also be due to increase glutathione oxidase activity, scavenging of ONOO-, which with decreased CuZnSOD mRNA in the FF of patients with PCOS [160] contribute to the reported decrease in H_2_O_2_. This may be due to nutritional zinc deficiency common in PCOS patients, causing an imbalance in the Zn/Cu ratio and subsequent malfunction of SOD. All indications then support the notion that in PCOS, ^•^OH and O_2_^•−^ are the specific ROS driving the disorder.

Because of this, and the known inflammatory state contributing and resulting from metabolic dysfunctions in PCOS, antioxidant therapy such as vitamins C and E, N-Acetylcysteine (NAC), Coenzyme Q (CoQ10), Alpha Lipoic Acid (ALA), omega 3, selenium, and melatonin, to name a few, have been studied in PCOS [161–163]. It has been concluded that although antioxidant supplementation proves beneficial in relieving several PCOS pathogenic parameters including overall oxidative stress, IR, androgen levels, and follicular maturation, research regarding dosage, long-term use, potential hazards to offspring growth and development, and individual disease profile of each patient must be expanded.

## Conclusion

In this work, we have reviewed the clinical and oxidative stress mechanism associated with anovulation in PCOS. We reviewed experimental and clinical data that suggest NO pathway and deficiency in PCOS is the ultimate factor in ovulatory dysfunction, chronic anovulation and poor oocyte quality in PCOS. NO has not been shown to influence the pulsatile hormone production in the preoptic nucleus where GnRH neurons originate. However, we have produced evidence that ADMA functions to competitively inhibit NOS by binding to the L-Arg binding site, which then increases free L-Arg bioavailability, allowing for consumption by arginase and NOS dysfunction with subsequent decrease in NO and increase in ADMA induced O2•− production. The resultant higher production of O_2_^•−^ from NOS dysfunction mediates DNA damage and impairs the antioxidant defense, which have been implicated as contributory factors for the development of PCOS. Therefore, as suggested by Li and collaborators [164] strategies that would increase DDAH1 activity in ovarian cells may provide a novel approach for ameliorating anovulation in PCOS.

Future research efforts should concentrate on the role of NO in the preoptic nucleus to determine whether it has an influence on frequency and amplitude of GnRH firing that coordinates FSH and LH production in the pituitary gland that culminate in ovulation. Until such studies are done, the authors propose treatment that that would increase DDAH1 activity in ovarian cells or decrease the expression of cGMP and cAMP that could increase NO production in PCOS. In addition, the use of potassium channel openers, an ADMA inhibitor or NO donor may prove beneficial to PCOS patients.

## Data Availability

Not applicable.

## Abbreviations

PCOS: Polycystic ovary syndrome
NO: nitric oxide
iNOS: inducible nitric oxide synthase
nNOS: neuronal nitric oxide synthase
eNOS: endothelial nitric oxide synthase
H_2_O_2_: hydrogen peroxide
ADMA: asymmetric dimethylarginine
DDAH1: dimethylarginine dimethylaminohydrolase 1
O_2_^•^: superoxide
SOD: superoxide dismutase
IR: insulin resistance
LH: Luteinizing hormone
FSH: follicle stimulating hormone
GnRH: gonadotropin-releasing hormone
cAMP: cyclic adenosine monophosphate
ROS: reactive oxygen species
^•^OH: hydroxyl radical
ONOO^-^: peroxynitrite
RNS: reactive nitrogen species
NO_2_^-^: nitrite
NO_3_^-^: nitrate
CC: clomiphene citrate
